# Mass Spectrometry Imaging Combined with Sparse Autoencoder Method Reveals Altered Phosphorylcholine Distribution in Imipramine Treated Wild-Type Mice Brains

**DOI:** 10.3390/ijms25147969

**Published:** 2024-07-21

**Authors:** Md Foyzur Rahman, Ariful Islam, Md. Monirul Islam, Md. Al Mamun, Lili Xu, Takumi Sakamoto, Tomohito Sato, Yutaka Takahashi, Tomoaki Kahyo, Satoka Aoyagi, Kozo Kaibuchi, Mitsutoshi Setou

**Affiliations:** 1 Department of Cellular and Molecular Anatomy, Hamamatsu University School of Medicine, 1-20-1 Handayama, Chuo-ku, Hamamatsu 431-3192, Shizuoka, Japan; 2 Preppers Co., Ltd., 1-20-1 Handayama, Chuo-ku, Hamamatsu 431-3192, Shizuoka, Japan; 3 Quantum Imaging Laboratory, Division of Research and Development in Photonics Technology/International Mass Imaging and Spatial Omics Center, Institute of Photonics Medicine, Hamamatsu University School of Medicine, 1-20-1 Handayama, Chuo-ku, Hamamatsu 431-3192, Shizuoka, Japan; 4 Faculty of Science and Technology, Seikei University, 3-3-1 Kichijoji-kitamachi, Musashino-shi 180-8633, Tokyo, Japan; 5 Division of Cell Biology, International Center for Brain Science, Fujita Health University, Toyoake 470-1192, Aichi, Japan; 6 International Mass Imaging and Spatial Omics Center, Institute of Photonics Medicine, Hamamatsu University School of Medicine, 1-20-1 Handayama, Chuo-ku, Hamamatsu 431-3192, Shizuoka, Japan

**Keywords:** pharmacokinetics, imipramine, phosphorylcholine, AP-MALDI-MSI, sparse autoencoder

## Abstract

Mass spectrometry imaging (MSI) is essential for visualizing drug distribution, metabolites, and significant biomolecules in pharmacokinetic studies. This study mainly focuses on imipramine, a tricyclic antidepressant that affects endogenous metabolite concentrations. The aim was to use atmospheric pressure matrix-assisted laser desorption/ionization (AP-MALDI)-MSI combined with different dimensionality reduction methods to examine the distribution and impact of imipramine on endogenous metabolites in the brains of treated wild-type mice. Brain sections from both control and imipramine-treated mice underwent AP-MALDI-MSI. Dimensionality reduction methods, including principal component analysis, multivariate curve resolution, and sparse autoencoder (SAE), were employed to extract valuable information from the MSI data. Only the SAE method identified phosphorylcholine (ChoP) as a potential marker distinguishing between the control and treated mice brains. Additionally, a significant decrease in ChoP accumulation was observed in the cerebellum, hypothalamus, thalamus, midbrain, caudate putamen, and striatum ventral regions of the treated mice brains. The application of dimensionality reduction methods, particularly the SAE method, to the AP-MALDI-MSI data is a novel approach for peak selection in AP-MALDI-MSI data analysis. This study revealed a significant decrease in ChoP in imipramine-treated mice brains.

## 1. Introduction

Mass spectrometry imaging (MSI) provides the mass spectra of hundreds and thousands of molecules [1], and atmospheric pressure matrix-assisted laser desorption/ionization mass spectrometry (AP-MALDI-MS) can measure intact drugs and their metabolites with spatial distribution [2]. It offers enhanced sensitivity and selectivity, allowing for the precise localization of compounds. Previously, we successfully detected drugs with high spatial resolution at higher speed with higher sensitivity by iMScope^TM^ QT, an AP-MALDI mass imaging microscope [3,4].

MSI data offers high-dimensional mass spectra alongside coordinate information. Peak patterns contain a vast amount of information but are challenging to understand. Mass spectra contain many biomolecular peaks and peaks with similar intensity distributions, but significantly different spatial distributions cannot be extracted efficiently within a short time. Therefore, dimensionality reduction methods serve as valuable tools for analyzing MSI data. Among these techniques, principal component analysis (PCA), multivariate curve resolution (MCR), and sparse autoencoder (SAE) have emerged as powerful methods for extracting meaningful patterns and structures from large-scale biological datasets [5,6,7].

PCA, a widely used dimensionality reduction technique in biology, analyzes data components to extract informative features from high-dimensional datasets, aiding in the differentiation of MALDI-time-of-flight (TOF) MS data [8,9,10,11]. MCR deconvolutes complex mixtures of chemical components and gives non-negative results [10]. When applied to desorption electrospray ionization (DESI) imaging mass spectrometer, the components of MCR showed important biological features such as anatomical structures of the rat brain [12]. Autoencoder is an unsupervised nonlinear dimensionality reduction method based on artificial neural networks, which is more suitable for analyzing mass image data containing nonlinear factors, such as matrix effects [13], than conventional multivariate analysis, such as PCA and MCR. In terms of quantitative analysis based on mass spectrum and image data by time-of-flight secondary ion mass spectrometry (TOF-SIMS), SAE was compared with PCA and MCR, and SAE showed superior results [7]. SAE retrieves features from the MALDI data of *Listeria* species and classifies them with high accuracy by combining other machine-learning algorithms [14]. Previously, such methods have been applied to the human-skin mass image data using TOF-SIMS [5,6] and SAE weights indicating the important ions for each compound.

Imipramine, a tricyclic antidepressant medication, is a model substance for the pharmacokinetics research of drugs and is metabolized almost entirely to desipramine [15]. It inhibits the reuptake of specific neurotransmitters, including serotonin and norepinephrine, into the presynaptic neurons [16,17]. In addition, imipramine affects the metabolism of phospholipids, including phosphatidylcholine, in cell membranes. It can alter the synthesis and degradation of these lipids. Studies suggest that imipramine may induce changes in the composition of phospholipids in the cell membrane, possibly influencing the turnover and levels of phosphorylcholine (ChoP) [18,19].

ChoP is the hydrophilic polar head group of some phospholipids, including phosphatidylcholine, sphingomyelin, etc., which is composed of a negatively charged phosphate bonded to a small, positively charged, choline group. Due to its atherogenic and proinflammatory effects, it can play a vital role in atherosclerosis, being one of the main oxLDL epitopes [20]. In addition, it is recognized as a critical modification of the surface structures of bacteria with roles in bacterial pathogenesis [21].

In this study, we aimed to apply PCA, MCR, and SAE to the AP-MALDI-MSI data of the imipramine-treated and untreated mice to extract characteristic peaks and to identify the analytes related to drug efficacy. Also, we aimed to examine the distribution and impact of imipramine on endogenous metabolites in the brains of the treated wild-type mice. A key aspect of our research is the innovative application of these dimensionality reduction methods, especially the SAE method, to the AP-MALDI-MSI data, leading to the detection of significant mass peaks that differentiate between the two groups of mice. Furthermore, the study provides new insights into the biochemical effects of imipramine on the brain by revealing changes in the spatial distribution of ChoP, which contributes to a better understanding of the drug’s pharmacological mechanisms and potential side effects. Overall, this research bridges the gap between advanced imaging technologies and computational analysis, offering a powerful tool for exploring and understanding the biochemical landscape of the brain in response to pharmaceutical interventions.

## 2. Results

### 2.1. Detection of Imipramine and Its Metabolites in the Mice Brains by AP-MALDI-MSI

**Figure 1:**
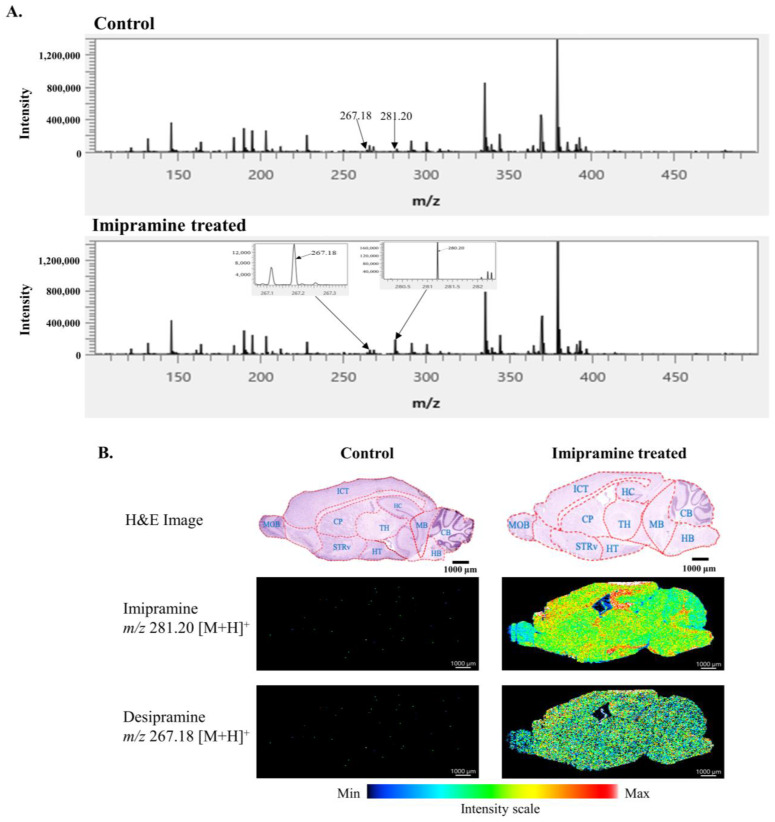
Distribution of imipramine and its metabolites in mice brains. (**A**) mass spectra showing peaks of imipramine at *m*/*z* 281.20 and its major metabolite desipramine at *m*/*z* 267.18 in the control and imipramine treated mice-brain sections at an *m*/*z* range of 100–500, and (**B**) H&E images of both control and imipramine treated whole mice brains (sagittal sectioning) and ion images of desipramine and imipramine distributions in both mice brains. CB: cerebellum, HT: hypothalamus, TH: thalamus, MB: midbrain, CP: caudate putamen, STRv: striatum ventral region, HB: Hindbrain, ICT: Isocortex, HC: Hippocampus and MOB: main olfactory bulb.

Imipramine and its metabolites were detected successfully in the imipramine-treated mice brains by AP-MALDI-MSI. As expected, the control mice showed no drug and its metabolites in the brains. Imipramine was detected with an *m*/*z* value of 281.20 [M + H]^+^, and its major metabolite, desipramine, was found at *m*/*z* 267.18 [M + H]^+^ in the imipramine-treated mice brains (Figure 1A). Although imipramine was distributed in most of the regions of the mice brains, however, it was localized mostly in the hippocampus, thalamus, hypothalamus, cerebellum, hindbrain, and iso-cortex regions. Like imipramine, desipramine was also distributed into all of the brain’s regions, mainly in the iso-cortex, hippocampus, thalamus, and hindbrain regions (Figure 1B).

### 2.2. Results of PCA, MCR, and SAE Analysis

**Figure 2:**
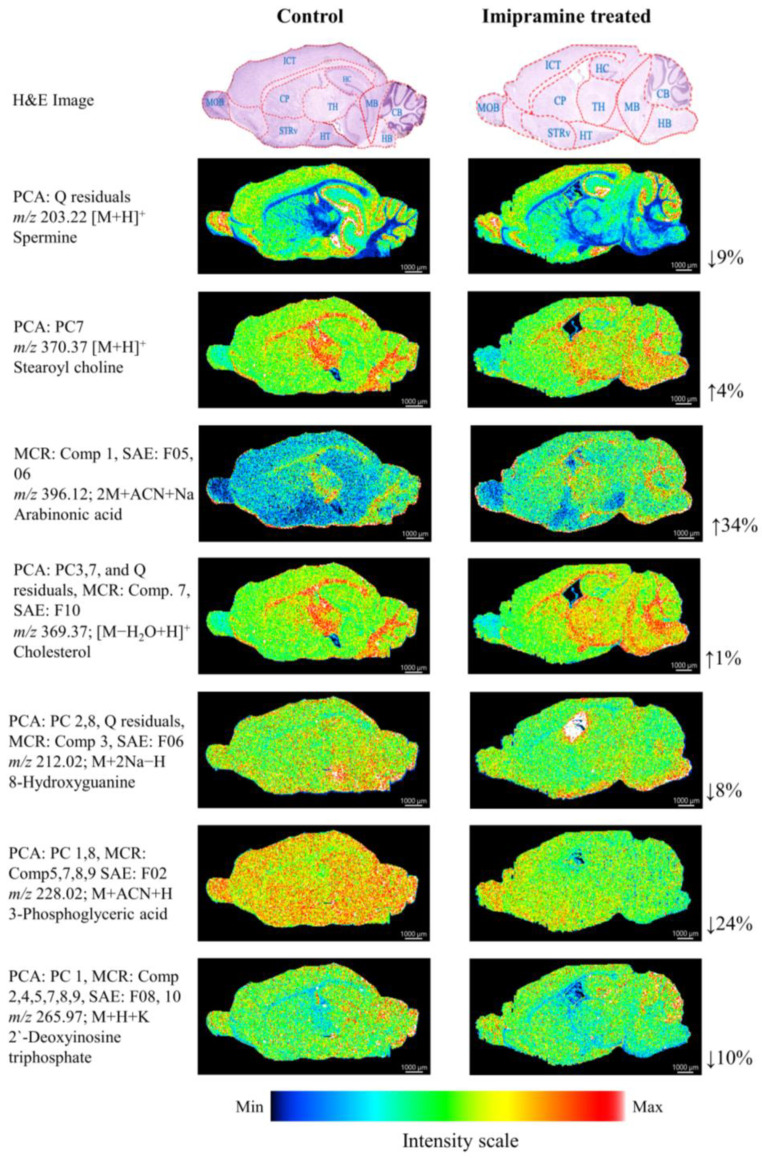
Results of PCA, MCR, and SAE analysis. In the PCA loadings only, *m*/*z* 203.22 and *m*/*z* 370.37 were identified as spermine and stearoyl choline as variable molecules, respectively. While the intensity of spermine was increased (9%) in the treatment, the intensity of stearoyl choline was decreased by only 4%. Both MCR and SAE methods found *m*/*z* 396.12, in which arabinonic acid was found as a candidate. The intensity of arabinonic acid was higher (34%) in the treatment. All three types of analysis methods showed *m*/*z* 369.37, 212.02, 228.02, and 265.97. Among these, the intensity of cholesterol (*m*/*z* 369.37) was found to be higher (1%) in the treatment group, whereas 8-hydroxyguanine, 3-phosphoglyceric acid, and 2’-deoxyinosine triphosphate showed lower intensities (8%, 24%, and 10%, respectively) in the same group. The percentage of intensity was measured by multiplying hundred with the result of the value of the average intensity of treatment divided by the average intensity of control. CB: cerebellum, HT: hypothalamus, TH: thalamus, MB: midbrain, CP: caudate putamen, STRv: striatum ventral region, HB: Hindbrain, ICT: Isocortex, HC: Hippocampus and MOB: main olfactory bulb.

We have assigned dimensionality reduction methods, including PCA, MCR, and SAE, to find the mass peaks associated with either bright and/or dark areas (Table S1). Eight *m*/*z* values, including *m*/*z* 203.22, 212.02, 228.02, 265.97, 369.37, 370.37, 396.12, and 184.07, were found to make high contributions to the features extracted by the PCA, MCR, and SAE methods. Meanwhile, others were non-significant, as they showed no signals (Figure 2, Table S1).

In the PCA method, Q residuals, which represent the rest of the principal components (PC10 to the last PC), showed that *m*/*z* 203.22 [M + H]^+^ identifies spermine, which was decreased overall by 9%, more specifically in the midbrain of the imipramine-treated mice. Stearoyl choline was identified by *m*/*z* 370.37 [M + H]^+^ and was listed in the mass peak of PC7, and the overall intensity level of stearoyl choline was higher (4%) in the treatment group than in the control group. However, in the thalamus, the distribution of stearoyl choline was lower in the imipramine-treated group, whereas, in the midbrain, the distribution was higher in the treatment group. Both MCR and SAE methods showed *m*/*z* 396.12 [2M + ACN + Na], which was identified as arabinonic acid with a higher difference (34%) between the two mice groups.

The PCA PC3, PC7, Q residuals, MCR Comp 7, and SAE F10 methods showed *m*/*z* 369.37 [M−H_2_O + H]^+^, which identifies cholesterol. The overall intensity level of cholesterol was higher (1%) in the treatment group than in the control. Specifically, cholesterol distribution was significantly higher in the midbrains of the imipramine-treated group. The PCA PC2, PC8, and Q residuals, MCR Comp 3, and SAE F06 methods showed *m*/*z* 212.02 [M + 2Na−H], which was identified as 8-hydroxyguanine and was found to be lower (8%) in the treatment. The PCA PC1, PC8, MCR Comp 5, 7, 8, and 9, and SAE F02 all showed *m*/*z* 228.02 [M + ACN + H], which was selected as 3-phosphoglyceric acid, and was decreased by 24% in the imipramine-treated group. In addition, PCA PC 1, MCR Comp 2, 4, 5, 7, 8, and 9, SAE F08, and F10 methods revealed *m*/*z* 265.97 [M + H+K], which was identified as 2’-deoxyinosine triphosphate and was lower (10%) in the treatment.

### 2.3. Distribution of Phosphorylcholine in the Mice Brains

**Figure 3:**
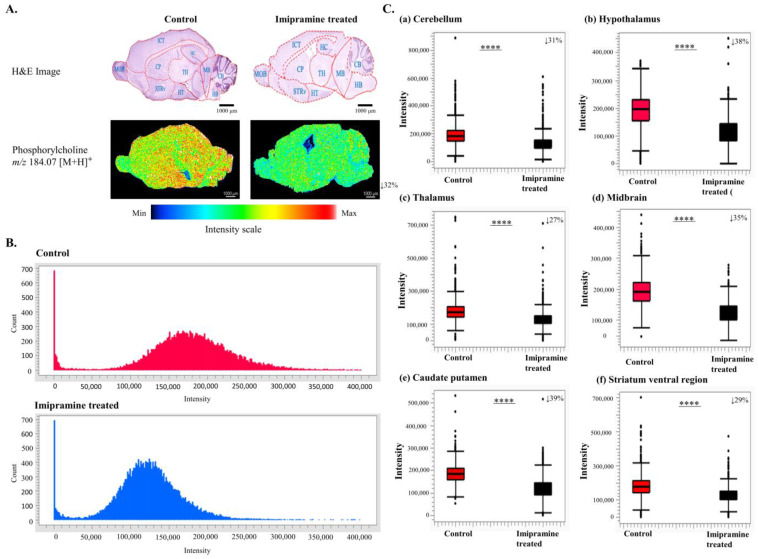
Phosphorylcholine distribution and comparison between the control and imipramine-treated groups. (**A**) H&E images showing the different regions of ChoP distribution in both control and imipramine-treated groups. The ion images present the ChoP distribution to compare the distribution between the groups. The ChoP level was decreased by 32% in the treatment. (**B**) histograms showing the differences in ChoP intensity between the two groups. Here, the intensity level of ChoP is significantly lower in the imipramine-treated mice brains. (**C**) Box plots illustrating the region-based intensity levels of ChoP distribution in both control and imipramine-treated mice brains. In all regions, including CB, HT, TH, MB, CP, and STRv, ChoP was found to be significantly lower by Welch’s *t* test (**** *p* < 0.0001) in the imipramine-treated mice brain than the control. The percentage decrease amounts (from (**a**) to (**f**) sequentially 31%, 385, 27%, 35%, 39%, and 29%) are shown in the upper-right corner of each figure. The percentage of intensity was measured by multiplying the hundred by the result of the value of the average intensity of treatment divided by the average intensity of control. CB: cerebellum, HT: hypothalamus, TH: thalamus, MB: midbrain, CP: caudate putamen, STRv: striatum ventral region, HB: Hindbrain, ICT: Isocortex, HC: Hippocampus and MOB: main olfactory bulb.

Among the three dimensionality reduction methods, only the SAE F02 showed *m*/*z* 184.07, which was identified as ChoP. That was changed to be the second highest (32% down; the highest change being the arabinonic acid at *m*/*z* 396.12, which was extracted by both MCR and SAE methods) between the control and treated groups’ mice brains (Figure 3A,B). 

In the imipramine-treated brain section, ChoP was distributed unevenly, with separated spots in specific brain regions including the cerebellum (CB), hypothalamus (HT), thalamus (TH), midbrain (MB), caudate putamen (CP), and striatum ventral region (STRv). The intensity level of the distribution of ChoP in all the regions of the brain was found to be significantly lower, by 31%, 385, 27%, 35%, 39%, and 29%, respectively, in the treated group (Figure 3C, Table S2).

## 3. Discussion

The MSI technique is well known for its high sensitivity, accuracy, and specificity in detecting drugs and analytes in biologics [1,22]. In pharmacokinetics study, the application of MSI techniques enables the simultaneous detection of both parent drugs and their metabolites, along with thousands of endogenous label-free molecules on tissue sections, all while preserving valuable information at nm to μm spatial resolution [23]. Therefore, MSI is widely used in pharmacokinetic studies, such as the distribution and metabolism of drugs in biological samples [24,25]. Drug distribution is a crucial process that involves exposing the target organs to the drug and is significantly affected by the amount of blood flow to these organs [26,27]. Studies of drug distribution are crucial for understanding the pharmacokinetics and the safety of the drug; therefore, research is being conducted continuously to advance drug-delivery systems [2].

In our study, we employed iMScope^TM^ QT (Shimadzu, Kyoto, Japan), an AP-MALDI mass imaging spectrometer, to reveal the effect of imipramine on mice brains. As expected, the tricyclic anti-depressant drug and its major metabolite, desipramine, were successfully detected in the treated mice at *m*/*z* 281.20 and *m*/*z* 267.18, respectively. In the previous study, we found a higher accumulation of imipramine in the thalamus, hypothalamus, septum, and hindbrain [2]. In our current study, we have found three more brain regions, namely the hippocampus, cerebellum, and iso-cortex, where a significantly higher accumulation of imipramine was observed. Like imipramine, desipramine distribution was observed all over the brain. However, the intensity level was not as high as imipramine. Desipramine was found comparatively higher in the iso-cortex, hippocampus, thalamus, and hindbrain regions of the treated group.

To extract the characteristic peaks associated with bright and/or dark areas of the ion images from the AP-MALDI-MSI data, we employed three-dimensionality reduction methods, including PCA, MCR, and SAE. PCA and SAE methods collected peaks from the bright and/or dark areas of the AP-MALDI imaging data, whereas the MCR method extracted masses associated with the bright areas of the images. All these methods extracted important masses from a large dataset by reducing the dimensions of the peaks (Table S1). Some mass peaks were associated with only one method, while most *m*/*z* values were correlated with two and/or three methods. Among the peaks obtained after analyzing the MSI data, we found eight *m*/*z* values that were significant for separating the imipramine-treated mice from the control group (Figure 2 and Figure 3A). From the PCA loadings, *m*/*z* 203.22 and *m*/*z* 370.37 were identified as significant, as they showed differences between the two groups. Both the MCR and SAE analysis found *m*/*z* 396.12. All three methods listed *m*/*z* 369.37, 212.02, 228.02, and 265.97, which were found significant for distinguishing between the treatment and control groups. From the SAE weights, *m*/*z* 184.07 [M + H]^+^, which identifies ChoP [28], is of importance for distinguishing between the control and imipramine-treated mice brains.

In our study, we found that imipramine treatment altered the distribution of ChoP. ChoP was found in both the control and imipramine-treated mice brains, but its intensity level was found to be significantly lower (*p* < 0.0001) both in the whole and in six specific regions of the imipramine-treated mice brains compared to the control mice brains (Figure 3). The six specific regions that showed decreased levels of ChoP after imipramine treatment are CB, HT, TH, MB, CP, and STRv (Figure 3C). ChoP, a significant component of the cell membrane, is integral to brain function through its presence in crucial phospholipids, such as phosphatidylcholine and sphingomyelin. It plays roles in membrane structure, neurotransmitter synthesis, signaling pathways, myelination, synaptic function, and neuroprotection [29,30]. These functions are essential for maintaining cognitive processes, neuronal communication, and overall brain health. Imipramine can affect ChoP levels by altering the metabolism of phospholipids, particularly phosphatidylcholine, and inducing lysosomal phospholipidosis [19,31]. In addition, it is known to inhibit the reuptake of neurotransmitters like serotonin and norepinephrine, which can lead to various downstream effects on cellular metabolism [32]. One of these effects can be the alteration of lipid metabolism, including the metabolism of phospholipids such as ChoP. Our research found a decreased level of ChoP as an effect of imipramine treatment. In addition, in this study, as a result, a unique m/z peak (*m*/*z* 184.07) was identified by the SAE method. This may be due to its capability of handling non-linear relationships, learning hierarchical features, processing large and complex datasets, providing architectural flexibility, and robustness to noise.

The study was constrained by a small sample size of only four mice (two per group). Additionally, the duration and dosage of imipramine treatment may not accurately reflect clinical conditions, limiting the generalizability of the findings. It remains uncertain whether the observed changes in the ChoP directly result from imipramine’s pharmacological effects or secondary consequences. These limitations should be acknowledged to offer a balanced interpretation of the research outcomes and guide future studies addressing these gaps. Future research should investigate the specific biochemical pathways through which imipramine affects ChoP distribution in the brain. Moreover, it is important to compare the effects of imipramine on ChoP distribution in wild-type mice with those in genetically modified mouse models, such as knockout mice for specific neurotransmitter receptors. Pharmaceutical companies will face a challenge in developing combination therapies that include imipramine and agents that stabilize ChoP levels. In addition, a study of potential biomarkers and mental parameters can be performed in the future. Also, another type of anti-depression drug, selective serotonin reuptake inhibitors (SSRIs), must be tested to investigate whether decreasing phosphorylcholine is related to the inhibition of serotonin incorporation. By addressing these future aspects, researchers can gain deeper insights into imipramine’s effects on brain biochemistry and potentially identify novel therapeutic targets for treating depression and other psychiatric disorders.

## 4. Materials and Methods

### 4.1. Chemicals and Materials

Powder of imipramine hydrochloride (Cas no: 113-52-0, purity > 98%, lot no: SKL0836, molecular weight: 316.87) and acetone (CAS no: 67-64-1, lot no: ACE6442, purity: >99%) were purchased from Fujifilm Wako Pure Chemical Industries (Osaka, Japan). We prepared normal saline water (0.9% solution of NaCl (CAS no: 7647-14-5, lot no: AGC4607, purity: 99.5%, Fujifilm Wako Pure Chemical Industries, Osaka, Japan) dissolved in MiliQ in our lab. Optimum Cutting Temperature (OCT) was purchased from Sakura Finetek, Tokyo, Japan. α-cyano-4-hydroxycinnamic acid (α-CHCA) matrix (CAS no: 28166-44-8; assay: ≥98% (TLC)) was purchased from Sigma-Aldrich (St. Louis, MO, USA). The staining compounds (hematoxylin and eosin), xylene, pathomount, LC-MS grade ultrapure water, and ethanol (purity: 99.5%) were purchased from Fujifilm Wako Pure Chemical Industries (Osaka, Japan).

### 4.2. Animals and Experimental Design

The experiment was performed using four wild-type (WT) C57BL/6J mice, bred in a standard animal facility (temperature:22 ± 2 °C, light–dark cycle: 12 h) of the Hamamatsu University School of Medicine, with a weight range of 28–31 g and an age of six months. At first, a pair of wild-type C57BL/6J mice (male and female) were bought from Japan SLC, Inc. and reared in the standard animal facility. The food for the mice was purchased from the PMI LabDiet^®^ Co., Ltd. (Howard Lake, MN, USA). It was mainly composed of protein (≥20%), fat (≥4.5%), and fiber (≥6.0%) and formulated for rodents. We did not add any supplements to the food. Our experimental mice were allowed to take this food ad libitum. The mice were then mated to breed more wild-type mice. From their offspring, we obtained our experimental mice. The mice were randomly divided into two groups: (i) control (normal saline treated; male, *n* = 2) and (ii) imipramine treated (male, *n* = 2). Imipramine (dissolved in normal saline water) was injected intraperitoneally (IP) at a dose of 30 mg/kg, while the control group was injected with normal saline water. After two hours of drug administration, all mice were euthanized by cervical dislocation. This procedure involves applying firm pressure at the base of the mouse’s skull while simultaneously pinching and twisting with the thumb and forefinger. Concurrently, the tail is pulled backward, which disconnects the spinal cord from the brain, leading to the mouse’s death. Later, the brain samples were quickly collected in dry ice, which was followed by storage in a −80 °C freezer until sectioning.

Our study was conducted according to the guidelines of the Institutional Animal Care and Use Committee (IACUC) of Hamamatsu University School of Medicine, Hamamatsu, Shizuoka, Japan, and all experimental procedures using living animals were approved by the institutional review board.

### 4.3. Preparation of Tissue Sections for AP-MALDI-MSI Measurements

We kept the frozen mice-brain samples, both the control and imipramine treated, inside the cryostat chamber of Leica CM1950 cryostat (Wetzlar, Germany) at −20 °C for 30 min to equilibrate the temperature for obtaining the optimal cutting condition and avoid damage to the tissues before the sectioning was performed. After that, the samples were mounted on round-shaped specimen discs using an OCT compound followed by the attachment on the object head for sectioning. After trimming at the optimum level, expected sagittal sections of the brains were collected at a thickness of 10 μm and then mounted on the pre-cooled indium tin oxide (ITO) coated glass slides (100 Ω, Matsunami, Osaka, Japan) by the thaw-mounted method. Before applying the matrix to the samples, the ITO-coated slides were dried at room temperature using a dry vacuum pump (ULVAC DTC-21) for 15 min. Then we applied a 0.7 μm thick layer of CHCA matrix onto the tissue sections using iMLayer^TM^ (Shimadzu, Japan) at 250 °C under vacuum conditions.

### 4.4. Acquisition of AP-MALDI-MSI by iMScope^TM^ QT

To obtain the MSI data, we placed the CHCA matrix-coated tissue samples in the vacuum chamber of the iMScope^TM^ QT, an imaging mass microscope (Shimadzu, Kyoto, Japan). The whole instrumental set comprised the following: an AP-MALDI source for ionization of the samples, a quadrupole time-of-flight (Q-TOF) mass spectrometer, and an integrated optical microscope for high spatial resolution imaging. Initially, the iMScope^TM^ QT parameters were optimized using standard drugs as follows: laser power of 60%, a laser diameter of 25 μm, a repetition number of 1000 Hz, the number of laser shots was 100, a detector voltage of 2.0 kV, the heat block temperature was 450 °C, and the DL temperature 250 °C. In addition, we conducted the AP-MALDI-MSI experiments in positive ion mode over the range of *m*/*z* 100–500.

### 4.5. Hematoxylin and Eosin (H&E) Staining and Scanning

After obtaining the MSI data, we performed hematoxylin and eosin (H&E) staining of the same brain-tissue sections to visualize the microanatomy of the samples. The samples were pre-washed for a few seconds with acetone to remove the CHCA matrix before the H&E staining was started. Then, the sample-containing ITO slides were placed into hematoxylin for 5 min, which was followed by washing with tap water for 3 min. Afterward, the slides were rinsed with 80% ethanol (80:20, ethanol:water (*v*/*v*)) for 1 min and, after that, submerged into eosin for 10–30 s to develop color. The slides were then washed with water for 1 min, which was followed by the dehydration of the tissue samples by passing them through a series of increasing ethanol concentrations (80%, 90% 100%, and 100%) for 1 min each time. Finally, xylene was used to remove any remaining water on the samples, and Pathomount, a mounting medium, was applied to the samples to protect the tissues from oxidation and to attach coverslips to the samples. Then, we used a NanoZoomer S60 (Hamamatsu Photonics, Hamamatsu, Japan) at 40×scanning mode for digital scanning and imaging of the H&E-stained tissue samples.

### 4.6. MSI Data Analysis

We employed IMAGEREVEAL^TM^ MS software (v. 1.30.0.11472, Shimadzu, Kyoto, Japan) to analyze the MSI data. First, we selected the region of interest (ROIs) of the brain sections. Then, we normalized the data by total ion current (TIC). During the analysis, we selected a threshold value of 0% and a tolerance/bin size of 0.01, while the other parameters remained default. There were 8047 mass peaks ranging from *m*/*z* 149.425301 to 551.725301 in the mass spectra of the samples. The mass images of the imipramine-treated sample (325 × 143 pixels) and the control sample (344 × 147 pixels) were merged into one dataset. The dataset was analyzed by PCA and MCR using PLS Toolbox (Eigenvector Research Inc., WA, USA) and, then, was analyzed by SAE using the Deep Learning Toolbox of Matlab (MathWorks, Inc., MA, USA). The SAE setting was similar to the previous study on the evaluation of human skin using TOF-SIMS [5].

Each of these three methods has strengths and is tailored for different data types and applications. The PCA excels with linear relationships and is computationally efficient, making it suitable for various applications. MCR yields highly interpretable results for complex mixtures, especially in chemical and spectroscopic analysis. SAE, capable of capturing non-linear relationships, is decisive for feature extraction and learning representations in complex datasets. We have demonstrated PCA score images, MCR concentration images, and SAE features images in Figure S1. In addition, we have listed PCA loadings, MCR spectrum matrices, and SAE weights in Table S1, indicating important peaks and making high contributions to the features extracted by each method.

We performed Welch’s *t*-test for the statistical significance analysis of the intensity values between the groups. The Welch’s *t*-test, also known as the unequal variance t-test, is a statistical test used to compare the means of two groups. It is a modification of the traditional Student’s *t*-test and is more reliable when the two samples have unequal variances, and the sample sizes may be unequal. The F-test can be used to test for chi-square (i.e., whether the variances of the two sets of data are equal), with *p* < 0.05 indicating that the variances are unequal. In our analysis, the F test *p* < 0.05 (Table S2) means variances are not equal, so we used Welch’s *t*-test. We used NDP.view 2 software (version U12388-01, Hamamatsu Photonics, Hamamatsu, Japan) to process and obtain the H&E images of the tissue samples.

## 5. Conclusions

The application of dimensionality reduction methods like PCA, MCR, and SAE to AP-MALDI-MSI data simplifies and accelerates the identification of important peaks in large MSI datasets. Using these methods on AP-MALDI-MSI data from mice-brain sections acquired with iMScopeTM QT, we identified several mass peaks that distinguished between two groups of mice. This demonstrates the effectiveness of integrating MSI with SAE techniques, highlighting how advanced data analysis enhances the detection and interpretation of molecular distributions in biological tissues. Notably, ChoP, which was identified only by the SAE method, was found to be an important biomolecule reduced in the brains of the imipramine-treated mice, providing new insights into the biochemical effects of imipramine, its pharmacological mechanisms, and potential side effects. This research advances the understanding of imipramine’s biochemical impact and improves imaging and data-analysis methodologies, influencing various biomedical research fields.

## Data Availability

The article includes all essential data, but additional supporting information can be obtained by submitting a written request to the corresponding author.

## Supplementary Materials

The following supporting information can be downloaded at https://www.mdpi.com/article/10.3390/ijms25147969/s1: Figure S1: Component images extracted by Principal Component Analysis (PCA) (A), Multivariate Curve Resolution (MCR) (B) and Sparse autoencoder (SAE) (C) for control and imipramine treated mice brains; Table S1: PCA loadings, MCR spectrum matrices, and SAE weights. Several *m*/*z* were found to make high contributions to the features extracted by PCA, MCR, and SAE. Table S2: *p*-values of different statistical tests. All sample regions show significant *p*-values on both the F-test and Welch’s *t*-test.
