# Supplementary material for: Mass Spectrometry Imaging Combined with Sparse Autoencoder Method Reveals Altered Phosphorylcholine Distribution in Imipramine Treated Wild-Type Mice Brains
Source: Int J Mol Sci. 2024 Jul 21;25(14):7969. doi: 10.3390/ijms25147969 (PMC11276679; doi:10.3390/ijms25147969)
Supplement: Supplementary file 1 [file ijms-25-07969-s001.zip › ijms-3095669-supplementary.pdf]

Supplementary Materials

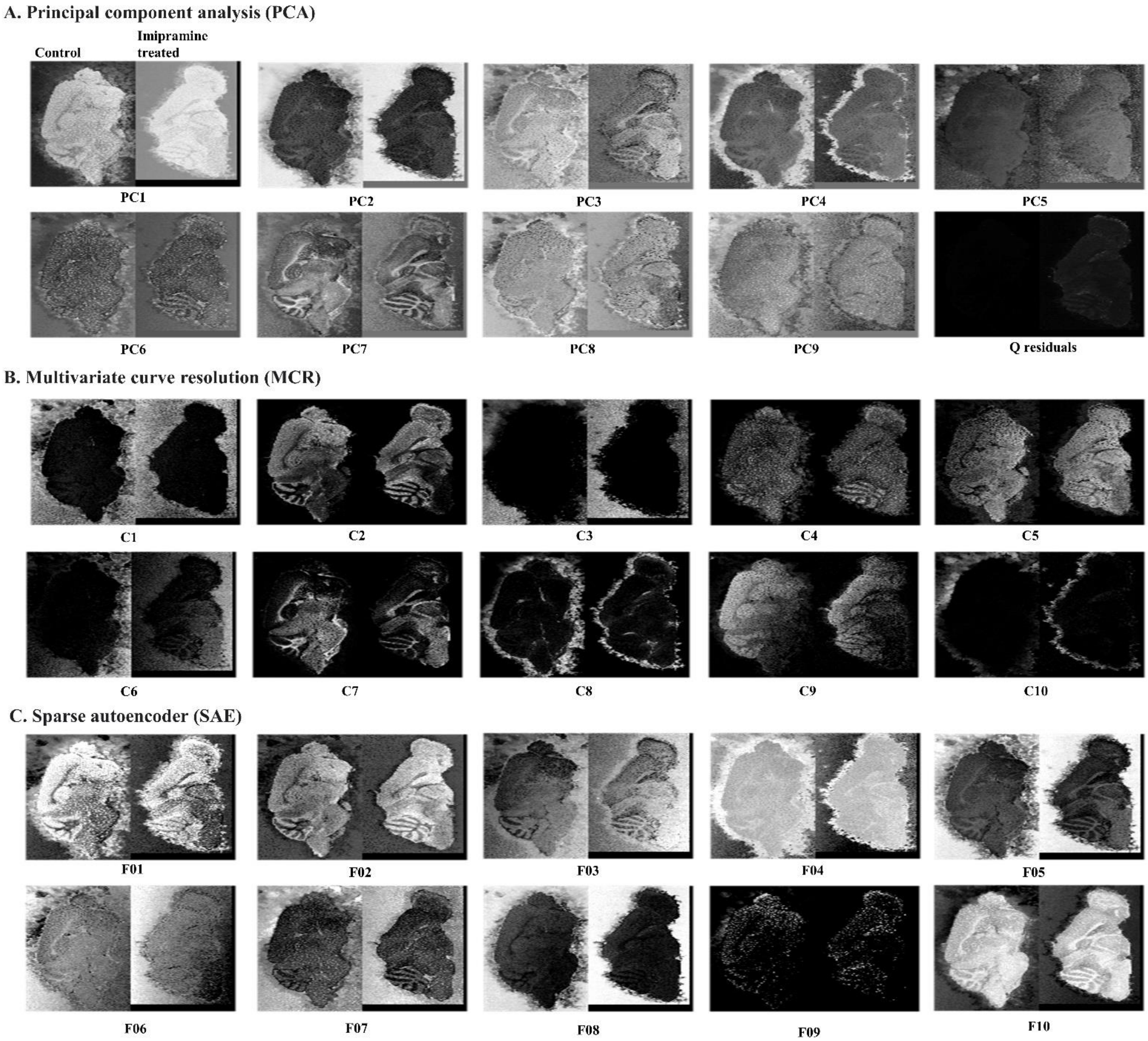

Figure S1: Component images extracted by Principal Component Analysis (PCA) (A), Multivariate Curve Resolution (MCR) (B) and Sparse autoencoder (SAE) (C) for control and imipramine treated mice brains. In each residue figure, the left image is the control mice brain section and the right image showing the imipramine treated brain section.

Table S1: PCA loadings, MCR spectrum metrices, and SAE weights. Several *m/z* were found making high contributions to the features extracted by PCA, MCR and SAE.

A. PCA: Mass peaks associated with bright areas

PC 1 (61.16%)	Mass	PC 2 (27.30%)	Mass	PC 3 (2.75%)	Mass	PC 4 (2.26%)	Mass	PC 5 (0.97%)	Mass	PC 6 (0.64%)	Mass	PC 7 (0.56%)	Mass	PC 8 (0.31%)	Mass	PC 9 (0.18%)	Mass	Q Residuals (3.87%)	Mass
4.56E-01	265.9753	2.93E-01	234.0253	4.89E-01	195.0753	4.10E-01	249.9753	3.52E-01	234.0253	3.93E-01	492.9753	3.67E-01	369.3753	3.89E-01	228.0253	3.42E-01	492.9753	5.86E+08	212.0253
3.02E-01	195.0753	2.38E-01	212.0253	3.16E-01	195.1253	3.39E-01	461.0253	2.82E-01	195.0753	2.15E-01	195.1253	2.14E-01	492.9753	2.27E-01	476.9753	1.92E-01	461.0253	4.65E+08	345.9253
2.69E-01	228.0253	2.23E-01	335.1253	2.70E-01	196.0753	2.22E-01	476.9753	2.76E-01	445.0253	2.14E-01	476.9753	1.73E-01	411.0253	1.94E-01	212.0253	1.82E-01	195.0753	4.25E+08	203.2253
2.02E-01	379.0753	2.19E-01	379.0753	1.73E-01	369.3753	2.06E-01	439.0253	2.46E-01	461.0253	1.71E-01	494.9753	1.64E-01	455.0253	1.93E-01	195.0753	1.43E-01	346.1253	4.12E+08	369.3753
2.01E-01	335.1253	1.86E-01	401.0753	1.27E-01	263.1253	1.44E-01	417.0753	1.98E-01	249.9753	1.58E-01	493.9753	1.56E-01	370.3753	1.42E-01	229.0253	1.42E-01	494.9753	3.95E+08	476.9753
1.95E-01	249.9753	1.74E-01	423.0753	1.27E-01	196.1253	1.31E-01	250.9753	1.55E-01	423.0753	1.17E-01	195.1753	1.54E-01	508.3253	1.04E-01	266.0253	1.30E-01	493.9753	3.93E+08	238.0753

Mass peaks associated with dark areas

PC 1 (61.16%)	Mass	PC 2 (27.30%)	Mass	PC 3 (2.75%)	Mass	PC 4 (2.26%)	Mass	PC 5 (0.97%)	Mass	PC 6 (0.64%)	Mass	PC 7 (0.56%)	Mass	PC 8 (0.31%)	Mass	PC 9 (0.18%)	Mass	Q Residuals (3.87%)	Mass
6.55E-06	153.8253	-3.85E-01	265.9753	-3.21E-01	249.9753	-2.26E-01	265.9753	-2.60E-01	335.1253	-3.24E-01	228.0253	-2.52E-01	345.9253	-2.43E-01	461.0253	-2.36E-01	476.9753	1.87E+04	153.8253
6.59E-06	154.3753	-1.86E-01	228.0253	-2.36E-01	234.0253	-2.18E-01	234.0253	-2.23E-01	379.0753	-2.08E-01	369.3753	-2.07E-01	195.1253	-2.39E-01	195.1253	-2.18E-01	379.0753	1.92E+04	154.6753
6.67E-06	154.6753	-1.65E-01	492.9753	-2.18E-01	461.0253	-2.09E-01	445.0253	-2.02E-01	379.1253	-1.83E-01	345.9253	-1.83E-01	212.0253	-2.36E-01	369.3753	-1.94E-01	423.0753	2.03E+04	153.3253
6.73E-06	154.8253	-1.52E-01	267.9753	-1.52E-01	265.9753	-1.87E-01	423.0753	-1.92E-01	396.1253	-1.30E-01	195.0753	-1.54E-01	249.9753	-1.78E-01	265.9753	-1.62E-01	195.1253	2.03E+04	155.2253
6.90E-06	154.5253	-1.28E-01	266.9753	-1.50E-01	445.0253	-1.73E-01	345.9253	-1.42E-01	336.1253	-1.21E-01	184.0753	-1.36E-01	228.0253	-1.71E-01	195.1753	-1.53E-01	445.0253	2.09E+04	155.8253
6.93E-06	155.2253	-1.09E-01	345.9253	-9.27E-02	250.9753	-1.33E-01	492.9753	-1.22E-01	346.1253	-1.14E-01	392.2753	-1.27E-01	301.9253	-1.42E-01	263.1253	-1.52E-01	417.0753	2.16E+04	166.3253

B. MCR: Mass peaks associated with bright areas

Comp. 1 (15.98%)	Mass	Comp. 2 (16.13%)	Mass	Comp. 3 (21.04%)	Mass	Comp. 4 (10.69%)	Mass	Comp. 5 (9.21%)	Mass	Comp. 6 (4.64%)	Mass	Comp. 7 (5.61%)	Mass	Comp. 8 (5.02%)	Mass	Comp. 9 (3.04%)	Mass	Comp. 10 (4.74%)	Mass
3.59E-01	335.1253	6.70E-01	265.9753	4.67E-01	234.0253	5.81E-01	265.9753	6.23E-01	195.0753	5.21E-01	195.1253	4.35E-01	195.0753	4.54E-01	249.9753	3.98E-01	379.0753	4.81E-01	249.9753
3.15E-01	379.0753	2.92E-01	345.9253	3.20E-01	445.0253	5.21E-01	492.9753	5.55E-01	228.0253	3.84E-01	195.0753	3.72E-01	369.3753	3.67E-01	476.9753	3.50E-01	228.0253	4.58E-01	461.0253
2.80E-01	379.1253	2.59E-01	267.9753	3.10E-01	212.0253	2.47E-01	267.9753	2.31E-01	265.9753	3.64E-01	196.0753	3.40E-01	265.9753	2.60E-01	265.9753	3.44E-01	335.1253	4.03E-01	234.0253
2.66E-01	396.1253	2.52E-01	249.9753	2.89E-01	423.0753	2.23E-01	494.9753	1.82E-01	196.0753	2.20E-01	263.1253	1.88E-01	228.0253	2.55E-01	228.0253	3.11E-01	265.9753	1.95E-01	445.0253

C. SAE: Mass peaks associated with either bright or dark areas

F01	Mass	F02	Mass	F03	Mass	F04	Mass	F05	Mass	F06	Mass	F07	Mass	F08	Mass	F09	Mass	F10	Mass
0.34985	492.9753	0.33361	345.9253	0.52461	195.1253	0.40492	439.0253	0.59079	396.1253	0.25298	396.1253	0.5452	234.0253	0.29546	265.9753	0.26428	345.9253	0.487	265.9753
0.26927	494.9753	0.2964	228.0253	0.49398	195.0753	0.35801	250.0253	0.5482	346.1253	0.21886	212.0253	0.53272	423.0753	0.28567	335.1253	0.2122	492.9753	0.46621	492.9753
0.2597	493.9753	0.27823	184.0753	0.48138	195.1753	0.34149	250.9753	0.41168	401.0753	0.1955	380.1253	0.48448	357.0753	0.23656	379.0753	0.195	301.9253	0.42269	369.3753
0.23428	221.9753	0.27486	301.9253	0.47229	196.0753	0.33776	417.0253	0.40902	380.1253	0.18717	379.1253	0.41862	401.0753	0.22516	450.1753	0.1899	494.9753	0.3751	494.9753
0.23262	267.9753	0.24689	229.0253	0.42866	196.1253	0.32187	417.0753	0.38866	380.0753	0.17577	380.0753	0.36003	446.0253	0.22325	397.1253	0.18553	493.9753	0.3681	493.9753

Table S2: p-values of different statistical tests. All sample regions are showing significant p-values in both F-test and Welch`s t-test.

**Sample regions**	**F-test (compare variance) (p-value)**	**Welch`s t-test (p-value)**
Cerebellum	<0.0001	<0.0001
Hypothalamus	<0.0001	<0.0001
Thalamus	<0.0001	<0.0001
Midbrain	<0.0001	<0.0001
Caudate Putamen	<0.0001	<0.0001
Striatum ventral region	<0.0001	<0.0001
